# 

**Published:** 1887-08

**Authors:** 


### CONTENTS.

ORIGINAL COMMUNICATIONS—

Alcoholic Liquors in the Practice of Medicine. By Eugene Foster, M. D., Augusta, Ga. . . . . . . . 325

Episcleritis. By R. O. Cotter, M. D., Macon, Ga. . . . . . . . 337

A Case of Malarial Manifestations due to Traumatism. By R. R. Harden, M. D., Harmony Grove, Ga. . . . . . . . 339

CLINIC REPORTS—

Multiple Neuritis. By T. M. Holmes, M. D., Rome, Ga. . . . . . . . 341

SOCIETY REPORTS—

Clinical Society of Maryland. . . . . . . . 347

Chicago Medical Society. . . . . . . . 353

CORRESPONDENCE.

Our New York Letter. . . . . . . . 357

Philadelphia Lying-in Charity. . . . . . . . 360

BOOK REVIEWS—

Text-book of Pathological Anatomy and Pathogenesis. By Ernest Zeigler. . . . . . . . 365

Wear and Tear; or, Hints for the Over-worked. By S. Weir Mitchell, M. D., LL. D. . . . . . . . 367

Practical Lessons in Nursing. By Charles K. Mills, M. D. . . . . . . . 367

The Practitioner's Handbook of Treatment; or, the Principles of Therapeutics. By J. Milner Fothergill, M. D. . . . . . . . 368

Transactions of the Associations of American Physicians. . . . . . . . 368

Earth as a Topical Application in Surgery. By Addinell Hewson, M. D. . . . . . . . 369

Drug Eruptions. By Prince A. Morrow, M. D. . . . . . . . 370

Medical Electricity. By Roberts Bortholow, M. D. . . . . . . . 371

A Manual of Treatment by Massage. By Joseph Schreiber, M. D. . . . . . . . 371

EDITORIAL—

The Candler Bill. . . . . . . . 373

International Medical Congress. . . . . . . . 374

Items. . . . . . . . 340, 376

